# Synergistic Activation of Peroxymonosulfate by CoMnOx Supported on Coal Gangue for Alkaline Wastewater Treatment

**DOI:** 10.3390/toxics14010029

**Published:** 2025-12-26

**Authors:** Ke An, Weiwei Yang, Houhu Zhang

**Affiliations:** 1 School of Chemical and Environmental Engineering, China University of Mining and Technology-Beijing, Beijing 100083, China; 2 Nanjing Institute of Environmental Sciences, Ministry of Ecology and Environment of the People’s Republic of China, Nanjing 210042, China

**Keywords:** coal chemical wastewater, phenol degradation, advanced oxidation processes (AOPs), peroxymonosulfate (PMS), bimetallic catalysts

## Abstract

This study explores the application of a cobalt–manganese oxide catalyst supported on coal gangue (CoMnOx@CG) for peroxymonosulfate (PMS) activation to degrade phenol in coal chemical wastewater (CCW). The synthesized CoMnOx@CG catalyst demonstrated remarkable catalytic activity, achieving above 90% phenol removal within 10 min at pH 9 and 11. More importantly, the catalyst exhibited excellent stability and reusability, maintaining over 85% phenol removal efficiency after four consecutive cycles and cobalt leaching below 100 μg/L. Quenching experiments and electron paramagnetic resonance (EPR) analyses revealed that singlet oxygen (^1^O_2_), sulfate radicals (SO_4_·^−^), and hydroxyl radicals (·OH) contributed to the degradation process. When treating actual CCW, the system significantly reduced both phenol and fluorescent dissolved organic matter, demonstrating its effectiveness for complex wastewater matrices. CoMnOx@CG provides a sustainable and practical solution for alkaline refractory wastewater remediation.

## 1. Introduction

China is the dominant country in the global coal chemical industry, consuming 120 million tons of coal and thereby generating about 3.50 × 10^8^ m^3^ of wastewater in 2020 [1,2]. The generated coal chemical wastewater (CCW) is a typical complex refractory wastewater, containing a large amount of phenols, polycyclic aromatic hydrocarbons (PAHs), nitrogenous heterocyclic compounds, long-chain alkanes, etc. [3,4,5]. Among them, phenol is one of the most abundant and dominant pollutants, capable of causing both acute and chronic health effects, ranging from skin, eye, and mucous membrane irritation [6,7,8]. Although conventional treatment of CCW usually integrates physicochemical pretreatment, biological processes, and advanced treatment technologies, the high toxicity and poor biodegradability of phenolic compounds often result in incomplete removal, thereby posing persistent risks to aquatic environments and human health [9,10].

To overcome these limitations, advanced oxidation processes (AOPs) have attracted increasing attention, as they exhibit extraordinary performance in degrading refractory phenolic compounds owing to their generation of diverse reactive oxygen species (ROS). Peroxymonosulfate (PMS) is commonly recognized as the superior source of ROS through different activation methods, such as heating [11,12,13], ultrasonic irradiation [14,15,16], ultraviolet light [17,18], and transition metals [19,20,21,22,23]. Among these methods, transition metals are regarded as promising candidates for PMS activation because they do not require continuous external energy input and are relatively easy to operate. Especially, cobalt-based catalysts are widely recognized as the most efficient PMS activators [24,25,26,27,28].

Despite their high activity, cobalt-based single-metal catalysts suffer from cobalt leaching, which leads to secondary pollution and compromised stability. To address this issue, multicomponent metal oxides have been developed. The strong interactions between the metal components in these systems effectively suppress cobalt leaching and enhance synergistic effects, thereby improving catalytic performance [29,30,31,32,33]. Among them, cobalt–manganese oxides, characterized by their multiple valence states, low toxicity, and the abundance of manganese resources, are considered efficient and stable catalysts for activating PMS [32]. Recent studies have shown that Co–Mn bimetallic oxides can significantly enhance PMS activation compared with single-metal oxides, due to the synergistic interactions between Co and Mn species. For instance, porous Mn/Co composite oxides have been reported to exhibit superior catalytic performance for PMS activation, which was attributed to enhanced redox cycling between multiple valence states and improved electron transfer efficiency [34]. Similarly, CoMn oxides loaded on SBA-15 have demonstrated improved catalytic activity toward the activation of PMS for the degradation of organic pollutants, suggesting that the integration of Co and Mn can optimize both the active sites and surface properties [35]. In addition, spinel-type Mn_x_Co_3-x_O_4_ materials have been demonstrated to promote PMS activation through improved electron transfer via redox cycles between Co^2+^/Co^3+^ and Mn^3+^/Mn^4+^ [36]. These comparative studies highlight the importance of Co–Mn synergistic effects in designing highly efficient PMS activation catalysts and justify further investigation into structurally optimized Co–Mn-based materials.

However, the practical application of high-performance powdered nano-catalysts is often hampered by their tendency to agglomerate, which reduces active sites, making them difficult to recover from aqueous solutions. While various support materials (such as graphene [37,38], carbon nanotubes [39,40], or zeolitic imidazolate framework [41,42]) have been developed to disperse catalysts and prevent aggregation, their widespread implementation is frequently constrained by complex synthesis procedures and high material costs. This underscores a critical need for developing cost-effective, efficient, and environmentally friendly support materials to bridge the gap between laboratory research and actual wastewater application.

Moreover, a key drawback of AOPs lies in their poor performance under strongly alkaline environments [30,43]. Although PMS activation systems generally achieve high catalytic efficiency at neutral or mildly alkaline pH, many types of CCW—for instance, post-oxidative effluents from bitumen production—are characterized by extreme alkalinity [44] (pH > 10). Neutralizing such effluents demands substantial acid input, which not only increases operational costs but also risks the emission of hazardous volatiles like hydrogen sulfide (H_2_S) [44,45,46]. To address the challenge of PMS activation under alkaline conditions, different strategies have been investigated. Heterogeneous metal oxides can activate PMS and generate sulfate and hydroxyl radicals, although performance often declines at very high pH [47,48]. Carbon-based composites such as biochar-supported transition-metal catalysts have also been shown to enhance PMS activation via electron transfer and radical generation mechanisms [49]. Despite these advances, most catalysts still exhibit reduced activity, stability issues, or complex synthesis under strongly alkaline conditions, indicating that further development of robust and low-cost systems remains necessary.

The large-scale accumulation of coal gangue (CG), representing roughly 15–20% of the annual coal production, poses many threats to the environment [50]. The stockpiling of coal gangue leads to spontaneous combustion, thus releasing many toxic gases, such as SO_2_, NO_x_, and CO [51]. Moreover, coal gangue harbors diverse heavy metals, which can easily be released into soils and groundwater [52]. Recently, activated coal gangue can serve as an effective support for Ni-based hydrogenation catalysts, where its improved surface properties promote the dispersion of active components and the synergistic interaction between metal and support, resulting in superior catalytic performance [53]. Moreover, abundant surface hydroxyl groups on coal gangue have been reported to facilitate persulfate adsorption and activation, indicating that coal gangue functions not merely as an inert support but also as an active participant in the catalytic process [54]. Therefore, the comprehensive utilization of coal gangue is of great significance. Although various carbon-based and metal–organic framework supports have been explored, the use of silicate-rich minerals as supports for transition-metal oxides in PMS activation has also attracted attention. Natural aluminosilicate minerals such as kaolinite, which are structurally similar to components of coal gangue, have been reported to directly activate peroxymonosulfate for the degradation of organic contaminants, owing to abundant surface hydroxyl groups and active sites that promote ROS generation [55]. Furthermore, recent studies have demonstrated that bimetallic Co–Mn oxides can effectively activate PMS when supported on silicate-derived materials. For example, CoMn_2_O_4_ spinel catalysts have been shown to activate PMS efficiently for pollutant degradation, indicating the promising catalytic performance of Co–Mn systems in PMS-based AOPs [56]. Similarly, Co–Mn oxide catalysts loaded onto fly-ash-derived SBA-15 (a silica/alumina-rich support) exhibited enhanced PMS activation for dye degradation, suggesting that integrating Co and Mn species with silicate supports can optimize active site dispersion and electron transfer during the activation process [35]. Given coal gangue’s intrinsic richness in SiO_2_ and Al_2_O_3_, these precedents support its rational design as an efficient, low-cost, and environmentally friendly support for CoMnOx catalysts in PMS oxidation systems.

In this study, bimetallic Co–Mn oxides supported on CG (CoMnOx@CG) were synthesized via a simple wet-impregnation method. Unlike conventional CoMnOx catalysts supported on synthetic materials, the proposed CoMnOx@CG utilizes coal gangue—a low-cost, silicate-rich industrial solid waste—as an active support. The intrinsic surface hydroxyl groups and abundant SiO_2_/Al_2_O_3_ components of coal gangue provide additional PMS-activating sites and enhance metal dispersion, enabling a waste-derived support to simultaneously improve catalytic efficiency and minimize catalyst cost. The as-prepared CoMnOx@CG was applied to activate PMS for the efficient degradation of phenol. Factors affecting the catalytic performance were systematically investigated, while the stability and reusability of the catalyst were also evaluated to assess its potential for practical applications. Furthermore, the possible catalytic mechanism of CoMnOx@CG was investigated using EPR tests and quenching experiments. This work not only proposes a cost-effective strategy for coal gangue utilization but also provides insights for developing sustainable catalysts for CCW remediation.

## 2. Materials and Methods

### 2.1. Materials

CG was obtained from a coal mine in Ordos, Inner Mongolia, China. Furfuryl alcohol (FFA, >98%) was supplied by TCI (Tokyo, Japan). tert-Butyl alcohol (TBA, >99.5%), ammonia solution (NH_3_·H_2_O, 28%), phenol, peroxymonosulfate (PMS, 2KHSO_5_·KHSO_4_·K_2_SO_4_), and 2,2,6,6-tetramethylpiperidine (TEMP) were purchased from Aladdin (Shanghai, China). Disodium hydrogen phosphate (Na_2_HPO_4_), ethanol (EtOH, >99.9%), hydrochloric acid (HCl), sodium hydroxide (NaOH), and cobalt nitrate hexahydrate (Co(NO_3_)_2_·6H_2_O) were obtained from Macklin (Shanghai, China). Methanol (MeOH, >99.9%) was provided by Thermo Fisher Scientific (Shanghai, China), sodium chloride (NaCl) by Mreda Technology (Beijing, China), and 5,5-dimethyl-1-pyrroline N-oxide (DMPO) by Dojindo (Kumamoto, Japan).

### 2.2. Characterization

The crystal structures of the synthesized materials were analyzed by X-ray diffraction (XRD, SmartLab SE, Rigaku, Tokyo, Japan). Surface morphology and elemental distribution were examined using scanning electron microscopy (SEM, Sigma 360, Carl Zeiss, Oberkochen, Germany) equipped with an energy-dispersive X-ray spectroscopy detector (EDS, Xplore 30, Oxford Instruments, Abingdon, UK). X-ray photoelectron spectroscopy (XPS) was conducted on a K-Alpha instrument (Thermo Fisher Scientific, Waltham, MA, USA) with Al Kα radiation. ROS were detected through electron paramagnetic resonance (EPR) using an EMXPLUS-6/1 spectrometer (Bruker, Billerica, MA, USA).

### 2.3. Preparation of CoMnOx@CG Catalyst

CoMnOx@CG was prepared through a wet-impregnation method (Figure S1). Briefly, coal gangue (CG, 30–70 mesh, 1.0 g) was dispersed in 30 mL of deionized water and ultrasonicated for 30 min. Subsequently, appropriate amounts of Co(NO_3_)_2_·6H_2_O and MnCl_2_ were added to achieve a Co:Mn atomic ratio of 1:1 in the precursor solution. The mixture was stirred until the metal salts were completely dissolved. The pH of the suspension was then adjusted to 9–10 using ammonia solution and maintained within this range throughout the subsequent 12 h stirring process to promote uniform deposition of Co and Mn species onto the CG surface. After filtration, the collected solid was dried at 60 °C for 6 h and then calcined in air at 350 °C for 6 h with a heating rate of 10 °C min^−1^. The final material was denoted as CoMnOx@CG.

### 2.4. Catalytic Degradation Experiments

The catalytic performance was assessed by assessing the degradation of phenol activated by PMS. In a typical run, 20 mg of catalyst was dispersed in 50 mL of phenol solution (20 mg/L, initial pH 9.0 ± 0.1) containing 100 mg/L PMS. At predetermined time intervals (0, 1.5, 3, 5, and 10 min), about 1.5 mL of solution was withdrawn with a syringe and immediately filtered through a 0.22 μm PTFE membrane. To quench ROS, high-concentration methanol solution was promptly added. Phenol concentration was analyzed at 510 nm using a UV-3000PC instrument (Mapada, Shanghai, China). The removal efficiency (η, %) and pseudo-first-order rate constant (k, min^−1^) are shown in Equations (1) and (2):

(1) $$\eta =\left(\frac{C_{0}-C_{t}}{C_{0}}\right)\times 100\%$$

(2) $$k=-\frac{1}{t}ln(\frac{C_{t}}{C_{0}})$$

Herein, C_0_ (mg/L) represents the initial phenol concentration, while Cₜ (mg/L) corresponds to the phenol concentration at a given reaction time t (min).

Three-dimensional excitation–emission matrix (3D-EEM) spectroscopy was performed using an F98 fluorescence spectrophotometer (Lengguang Technology, Shanghai, China) to investigate the changes in fluorescence characteristics. Metal leaching was quantified by inductively coupled plasma mass spectrometry (ICP-MS, iCAP Pro, Thermo Fisher Scientific, Waltham, MA, USA). The actual wastewater sample was derived from a coking industry effluent.

All catalytic degradation experiments were performed in duplicate unless otherwise stated. The reported values represent the mean of two independent experiments (n = 2), and the error bars shown in the figures indicate the standard deviation.

## 3. Results and Discussion

### 3.1. Characterization

**Figure 1:**
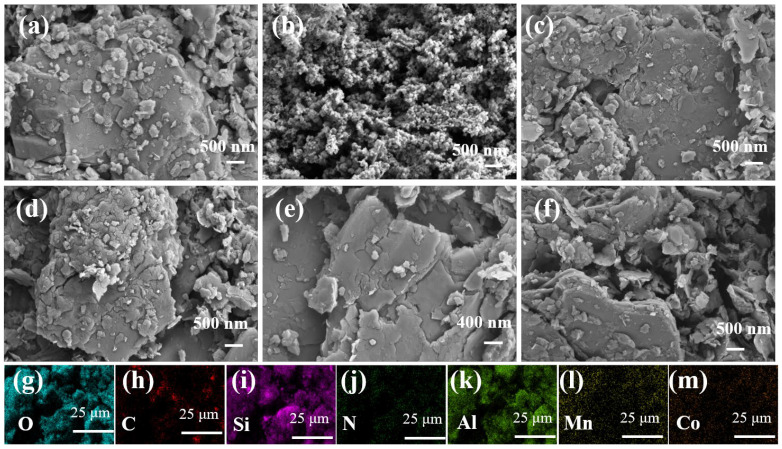
SEM images of (**a**) coal gangue; (**b**) CoMnOx; (**c**–**f**) CoMnOx@CG; (**g**–**m**) Elemental mapping of O, C, Si, N, Al, Mn, and Co.

As shown in Figure 1 and Figure S2, CG exhibited a relatively smooth surface in some regions, while other areas showed a densely stacked structure. These structural features of CG likely contribute to the uniform dispersion of CoMnOx, as the surface hydroxyl groups on CG facilitate the even distribution of CoMnOx nanoparticles, preventing aggregation. In contrast, the pristine CoMnOx displayed irregular and granular morphology with obvious agglomeration and compact stacking features. After loading CoMnOx onto CG (Figure 1c–f), the aggregation was effectively reduced, and the particles were more uniformly dispersed on the support. The EDS spectra confirmed the presence of O, C, Si, N, Al, Mn, and Co (Figure 1g–m). Elemental composition analysis indicated that the atomic ratio of Co to Mn was nearly 1:1, with contents of 3.12 wt% and 2.95 wt%, respectively. (Figures S3 and S4 and Table S1).

**Figure 2:**
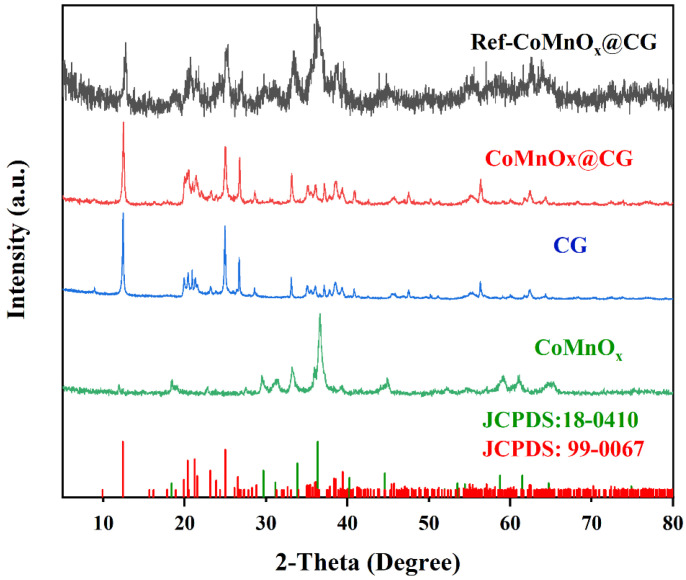
XRD patterns of the samples.

Figure 2 shows the XRD patterns of different samples. The diffraction peaks of CG are mainly distributed at 2θ = 12.41°, 20.38°, 21.23°, 23.13°, and 24.97°, which correspond to the Bragg planes of (001), (002), (−110), (−1−11), and (0−21), respectively (JCPDS: 99-0067). For the CoMnOx@CG sample, its diffraction pattern is nearly identical to that of CG, and the characteristic peaks of CoMnOx cannot be clearly observed. This is attributed to the low mass fraction of CoMnOx (Table S1), which results in its diffraction signals being overlapped by the strong peaks of CG.

To further confirm the formation of CoMnOx, a high CoMnOx loading sample (using 1 g CG, 2 g Co(NO_3_)_2_·6H_2_O and 0.866 g MnCl_2_ as precursors) was synthesized as a reference. As shown in the XRD pattern of Ref-CoMnOx@CG (Figure 2), the characteristic diffraction peaks of CoMnOx are observed at 2θ values of 33.82°, 36.33°, 44.58°, 58.72°, and 61.48°, which can be indexed to the Bragg planes of (113), (311), (400), (511), and (404), respectively (JCPDS: 18-0410). These results confirm the successful formation of the crystalline CoMnOx phase. Hence, although the diffraction peaks of CoMnOx cannot be clearly distinguished in CoMnOx @CG due to its low loading, the results from the reference sample provide strong evidence that CoMnOx has been successfully loaded onto the coal gangue support.

**Figure 3:**
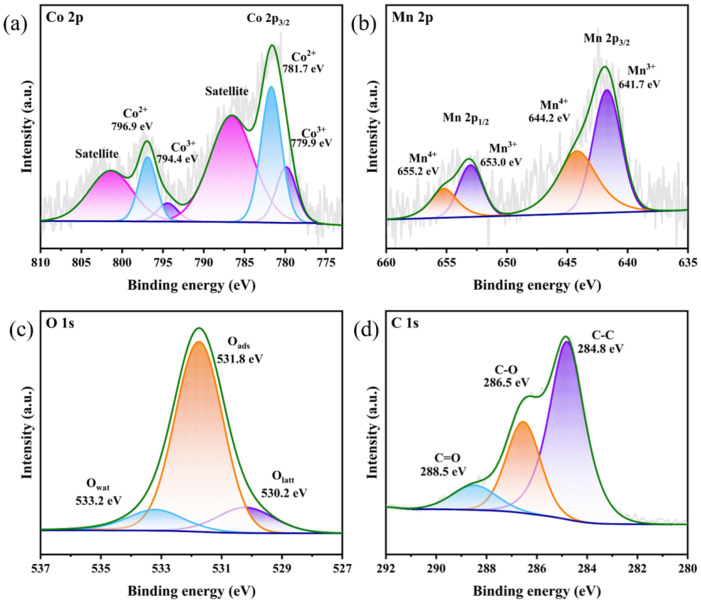
XPS spectra of Co 2p (**a**), Mn 2p (**b**), O1s (**c**) and C1s (**d**) for CoMnOx@CG.

XPS analysis was employed to investigate the surface elemental composition and chemical states of the samples. As shown in Figure S5, the survey spectrum confirmed the presence of Co, Mn, O, and C elements on the surface of CoMnOx@CG. The high-resolution spectra of Co 2p and Mn 2p are displayed in Figure 3. In the Co 2p spectrum (Figure 3a), two distinct spin–orbit double peaks corresponding to Co 2p_3/2_ and Co 2p_1/2_ are observed. The Co 2p_3/2_ components at 779.9 eV and 781.7 eV are assigned to Co^3+^ and Co^2+^, respectively, together with a shake-up satellite at ~786.6 eV, confirming the coexistence of mixed Co oxidation states [57,58,59].

The Mn 2p spectrum (Figure 3b) shows a broad Mn 2p_3_/_2_ feature containing two components centered at ~641.7 eV and ~643.8–644.2 eV, corresponding to Mn(III) and Mn(IV), consistent with the ranges reported in the literature [60,61]. The slightly higher binding energy of the Mn(IV) component relative to pure OMS-2 is attributed to the modified local coordination environment and Mn–Co interactions in the composite. Considering the pronounced multiplet splitting and satellite structure of transition-metal 2p spectra, the present deconvolution is used only for qualitative identification of oxidation states, and no quantitative Mn^3+^/Mn^4+^ or Co^2+^/Co^3+^ ratios are extracted. The O 1s spectrum (Figure 3c) can be deconvoluted into three components centered at 530.2 eV (lattice oxygen), 531.8 eV (defective or surface-adsorbed oxygen species), and 533.2 eV, which is attributed to adsorbed molecular water or weakly bound oxygen species [62,63]. These results suggest that the interaction between CoMnOx and coal gangue leads to enhanced electron transfer and defect formation, both of which are crucial for improving catalytic activity in PMS activation. The C 1s spectrum reveals C–C (284.8 eV), C–O (286.5 eV), and C=O (288.5 eV) species.

### 3.2. Catalytic Performance Evaluation

#### 3.2.1. Degradation Performance of Different Catalysts

**Figure 4:**
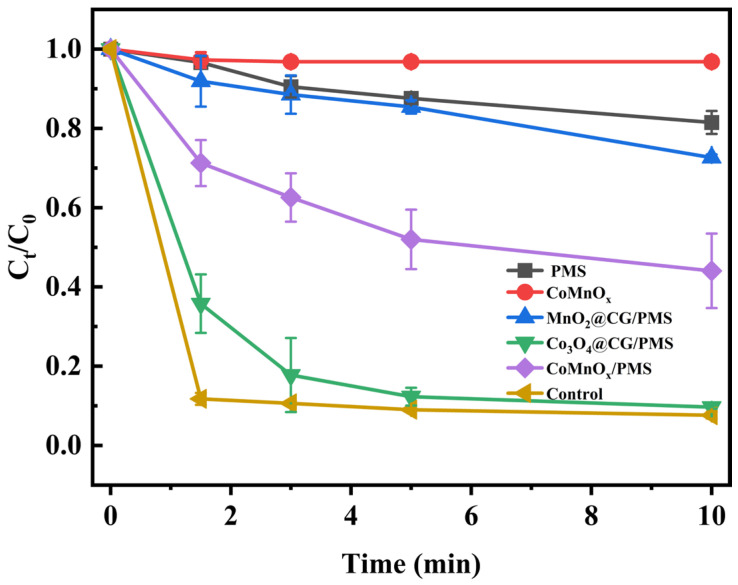
Comparison of phenol degradation efficiency under different systems. Control refers to the CoMnOx@CG/PMS system under identical reaction conditions (pH = 9, [PMS]_0_ = 100 mg/L, [phenol]_0_ = 20 mg/L, catalyst dosage = 400 mg/L, T = 298 K).

The catalytic performances of different catalysts for phenol degradation were evaluated, as shown in Figure 4. In the absence of catalyst (with PMS alone), phenol removal was only 18.5% after 10 min, confirming the weak oxidation capacity of PMS alone. Although phenol itself can partially activate PMS through quinone intermediates to generate ^1^O_2_ under alkaline conditions [64], this self-catalytic pathway was not sufficient for effective removal within such a short reaction period. With MnO_2_@CG, the removal efficiency was slightly improved but still limited at 27.4%. The unsupported CoMnOx exhibited higher activity with a removal efficiency of 55.9%. However, its performance was restricted by the agglomeration of active particles. In comparison, Co_3_O_4_@CG showed markedly enhanced activity, achieving 90.3% removal. Notably, CoMnOx@CG delivered the highest efficiency of 92.4%, which can be attributed to the synergistic effect between Co and Mn species and the stabilizing role of the coal gangue support, both of which collectively enable more effective PMS activation [65]. The apparent rate constant of CoMnOx@CG for phenol degradation was determined to be k = 0.26 min^−1^, indicating a much higher catalytic activity compared with these previously reported systems (Table S2).

#### 3.2.2. Effect of pH

**Figure 5:**
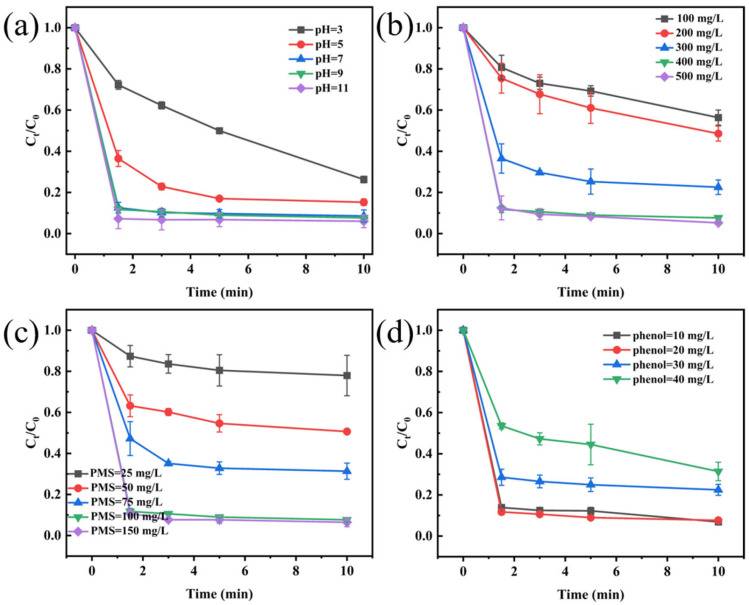
Degradation curves of CoMnOx@CG with (**a**) different initial pH values, (**b**) different catalyst dosages, (**c**) different PMS dosages, and (**d**) different initial phenol concentrations. General experimental condition: C_0_ (phenol) = 20 mg/L, C_0_ (PMS) = 100 mg/L, C_0_ (catalyst) = 400 mg/L, initial pH = 9.0, and T = 298 K.

The effect of initial pH on phenol removal in the CoMnOx@CG/PMS system was investigated, as shown in Figure 5a. The removal efficiency was only 73.7% at pH 3 after 10 min reaction, indicating that excessive H^+^ not only inhibited the formation of the key intermediate CoOH^+^ (vital for PMS activation) but also quenched the generated SO_4_·^−^ and ·OH radicals, thereby further suppressing the overall oxidative performance [66,67,68]. When the pH increased to 5 and 7, the removal efficiencies improved to 84.7% and 91.4%, respectively, suggesting that higher pH facilitated the generation of ROS. Under alkaline conditions, the system exhibited superior performance, achieving 92.4% and 93.9% removal at pH 9 and 11, respectively. These results indicate that CoMnOx@CG maintains high catalytic activity across a wide pH range, particularly in alkaline environments. Considering that CCW is typically alkaline, subsequent experiments were conducted at pH 9 to better simulate practical application scenarios.

#### 3.2.3. Effect of Catalyst Dosages

The catalyst dosage significantly influences the activation of PMS. As shown in Figure 5b, a low dosage of 100 mg/L resulted in a phenol removal efficiency of only 43.7% within 10 min. As the dosage increased to 200 mg/L and 300 mg/L, the removal rates improved to 51.4% and 77.5%, respectively. This enhancement can be attributed to the increased availability of active sites at higher catalyst dosages, which promotes PMS activation and ROS generation. A further increase in dosage to 400 mg/L and 500 mg/L led to excellent removal performances of 92.4% and 94.8%, respectively. However, beyond 400 mg/L, the increase in removal efficiency became less pronounced, which is possibly due to ROS self-quenching [69,70]. Consequently, a dosage of 400 mg/L was selected as the optimal condition to balance high removal performance with cost-effectiveness.

#### 3.2.4. Effect of PMS Dosages

The degradation of phenol exhibited pronounced dependence on PMS concentration (Figure 5c). At 25 mg/L PMS, the removal efficiency was only 22.1%, indicating that insufficient PMS dosage limited the generation of ROS and resulted in low degradation efficiency. With the increase in PMS concentration, the removal efficiency gradually improved, reaching 92.4% at 100 mg/L, which represented the optimal condition. However, when PMS concentration was further increased to 150 mg/L, the removal efficiency only slightly increased to 93.5%, suggesting that the system had approached its optimum and that excessive PMS did not significantly enhance the degradation. This is because an excessive amount of PMS can act as a scavenger of sulfate radicals, leading to the formation of less reactive SO_5_·^−^ species compared to SO_4_·^−^ [71].

#### 3.2.5. Effect of Phenol Dosages

The effect of initial phenol concentration (10–40 mg/L) on the degradation efficiency was evaluated in the CoMnOx@CG/PMS system. As shown in Figure 5c, an increase in the initial concentration from 10 mg/L to 20 mg/L resulted in a negligible change in removal efficiency (from 93.1% to 92.4%), indicating that the system maintained high performance within this concentration range. However, a further increase to 30 mg/L and 40 mg/L led to a notable decline in removal efficiency to 77.5% and 68.6%, respectively. Given that excellent performance was still achieved at 20 mg, a concentration more representative of phenolic pollutant levels in real wastewater, this level was selected as the benchmark for subsequent investigations.

### 3.3. Influences of Water Background Compounds

**Figure 6:**
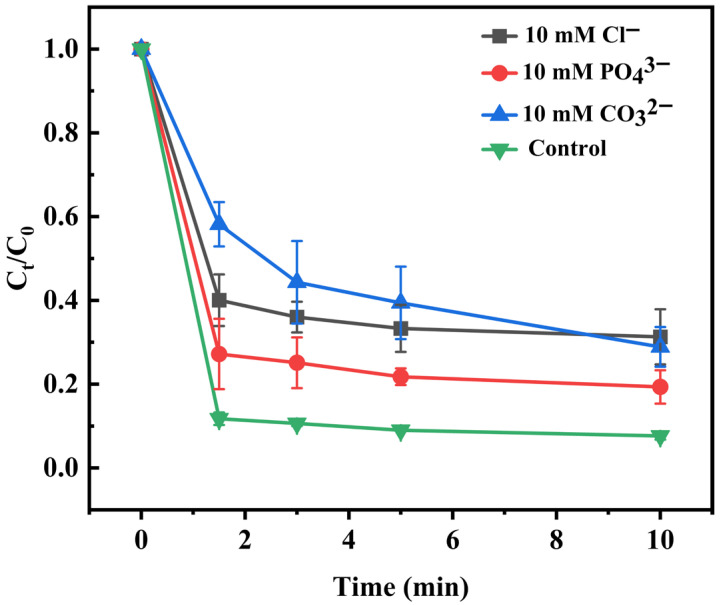
Degradation curves of CoMnOx@CG under different inorganic anions.

The impacts of different inorganic anions were investigated, as shown in Figure 6. The system’s removal efficiency decreased from 92.4% to 71.1% with the addition of CO_3_^2−^. This is because a portion of SO_4_·^−^ and ·OH reacts with CO_3_^2−^ to generate carbonate radicals (CO_3_·^−^), which exhibit lower reactivity and oxidation potential compared with SO_4_·^−^ and ·OH [72,73].

In the presence of 10 mM chloride, SO_4_·^−^ can react with Cl^−^ to produce chlorine radicals (Cl·) or dichloride radical anions (Cl_2_·^−^) [74,75]. However, these chlorine species have lower oxidation potentials (Cl· ≈ 2.4 V; Cl_2_·^−^ ≈ 2.1 V) compared to SO_4_·^−^ (2.6 V) and ·OH (2.8 V) [76], and their reaction rate constants with phenol are relatively small [77,78]. Therefore, although chloride introduces additional oxidative species, their contribution cannot compensate for the consumption of SO_4_·^−^ and ·OH. As a result, the overall degradation efficiency decreases in the presence of Cl^−^.

The addition of PO_4_^3−^ to the system resulted in a decrease in the removal efficiency from 92.4% to 80.7%. This is because PO_4_^3−^ can strongly adsorb onto the surface of CoMnOx and form inner-sphere complexes with the catalyst, thereby inhibiting PMS activation and decreasing degradation efficiency [79,80,81].

In contrast, singlet oxygen (^1^O_2_) formed during PMS activation is less susceptible to scavenging by phosphate and sulfate anions and may thus represent a relatively stable ROS contributor in the system, although it cannot fully compensate for the loss of SO_4_·^−^/·OH activity [82,83].

### 3.4. Mechanism of CoMnOx@CG

**Figure 7:**
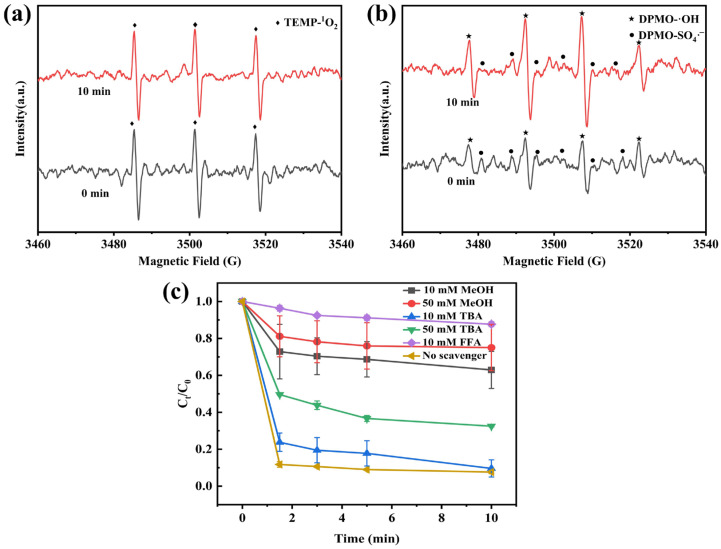
EPR spectra (**a**,**b**) and effect of scavengers on the degradation process (**c**).

EPR measurements were further employed to identify the ROS in the CoMnOx@CG/PMS system (Figure 7). The EPR spectrum exhibited characteristic 1:1:1 triplet signals attributed to ^1^O_2_, which confirms that ^1^O_2_ was involved in phenol degradation. Signals for DMPO- SO_4_·^−^ and DMPO-·OH adducts were also detected, indicating the concurrent presence of SO_4_·^−^ and ·OH in the system.

The quenching experiments further supported this conclusion. FFA, as a specific scavenger for singlet oxygen (^1^O_2_), significantly inhibited phenol degradation (FFA, k^1^O_2_ = 1.2 × 10^8^ M^−1^ s^−1^) [84,85], reducing the efficiency to only 12.3%. This verifies the critical role of ^1^O_2_ in the CoMnOx@CG/PMS system. Additionally, TBA is a specific scavenger for ·OH (TBA, k·OH = 3.8–7.6 × 10^8^ M^−1^ s^−1^), while MeOH acts as a scavenger for both ·OH and SO_4_·^−^ (MeOH, kSO_4_·^−^ = 1.6–7.8 × 10^7^ M^−1^ s^−1^, k·OH = 1.2–1.8 × 10^9^ M^−1^ s^−1^) [85]. Upon addition of 50 mM TBA, the phenol removal efficiency was 67.5%, whereas with 50 mM MeOH, the phenol removal efficiency decreased to 24.9%. Based on the apparent inhibition efficiencies derived from the C/C_0_ values, the relative contribution of different reactive oxygen species follows the order: ^1^O_2_ > SO_4_·^−^ > ·OH, indicating that singlet oxygen is the dominant oxidizing species in the CoMnOx@CG/PMS system. The negligible inhibition at 10 mM TBA further suggests that ·OH plays only a minor role, while the increasing inhibition with higher MeOH concentrations highlights the significant contribution of sulfate radicals. These findings demonstrate that multiple ROS, including ^1^O_2_, SO_4_·^−^, and ·OH, were simultaneously generated in the CoMnOx@CG/PMS system and collectively contributed to phenol degradation.

Co^2+^ and Mn^3+^ react with PMS to produce Co^3+^ and Mn^4+^, simultaneously generating SO_4_^2−^ and ·OH (Equations (1)–(3)) [28]. SO_4_·^−^ can convert into ·OH [86] (Equation (6)). Subsequently, Co^3+^ is reduced back to Co^2+^ by PMS, which is converted to SO_5_·^−^ (Equation (7)). The regenerated Co^2+^ species can then continuously activate PMS to produce ROS, thereby facilitating the degradation of phenol. However, the reduction of Mn^4+^ relied on the assistance of Co^2+^, because Mn^4+^ has a lower standard reduction potential and cannot be directly reduced to Mn^3+^ by PMS [87] (Equation (8)). In addition, the ^1^O_2_ can be produced through PMS self-decomposition, while SO_5_·^−^ further contributes to ^1^O_2_ formation [88] (Equations (9) and (10)). These cooperative radical and non-radical pathways generate ROS, which play a crucial role in the degradation of phenol.

After the reaction, XPS analysis of the spent CoMnOx@CG catalyst (Figure S6) shows changes in the surface chemical states of Co and Mn. The Co^2+^ content appears to increase, while the Mn^3+^/Mn^4+^ ratio shows no significant change within experimental error. This observation is consistent with the proposed electron transfer processes between Co and Mn species, in which Mn^3+^ is oxidized to Mn^4+^ (Equations (5) and (8)). Due to the signal-to-noise ratio of the XPS data, only substantial changes in oxidation states can be reliably detected; therefore, small variations cannot be confirmed.

Co^2+^ + HSO_5_^−^ → Co^3+^ + SO_4_·^−^ + OH^−^ (3)

Co^2+^ + HSO_5_^−^ → Co^3+^ + SO_4_^2−^+ ·OH (4)

Mn^3+^ + HSO_5_^−^ → Mn^4+^ + SO_4_·^−^ + OH^−^ (5)

SO_4_·^−^ + H_2_O →·OH + SO_4_^2−^ + H^+^ (6)

Co^3+^ + HSO_5_^−^ → Co^2+^ + SO_5_·^−^ + H^+^ (7)

Mn^3+^ + Co^3+^ → Mn^4+^ + Co^2+^ (8)

SO_5_·^−^ + H_2_O → HSO_4_^−^ + ^1^O_2_ (9)

HSO_5_^−^ + SO_5_^2−^ → HSO_4_^−^ + SO_4_^2−^ + ^1^O_2_ (10)

Compared with traditional single-metal PMS activation pathways, recent studies increasingly emphasize both radical (SO_4_·^−^, ·OH) and non-radical (^1^O_2_) pathways in heterogeneous and bimetallic systems. For instance, in spinel-type Co–Mn oxides such as Co_2_Mn_1_O_4_ and CoMn_2_O_4_, EPR and radical scavenging experiments have confirmed the coexistence of multiple ROS, including sulfate radicals and singlet oxygen, indicating that synergistic redox cycles between Co and Mn sites can enhance ^1^O_2_ production alongside conventional radical species, leading to improved pollutant degradation efficiency across a broad pH range [89,90]. Similarly, other mixed-metal oxides, such as CuO–CeO_2_, have been reported to favor singlet oxygen generation as the dominant PMS activation pathway, demonstrating the viability of non-radical routes in heterogeneous catalysis [82,91]. Furthermore, recent reviews highlight that ^1^O_2_ formation is not a marginal side reaction but can be actively promoted by deliberate catalyst design, such as tuning surface electronic structures and redox cycles [82].

Overall, the ROS profile observed in the CoMnOx@CG/PMS system, where ^1^O_2_ plays a critical role alongside SO_4_·^−^ and ·OH, aligns with emerging trends in PMS activation, emphasizing cooperative radical/non-radical mechanisms. This cooperative pathway likely contributes to enhanced phenol degradation efficiency, complementarity in reactivity, and resilience against common ROS scavengers in aqueous matrices.

### 3.5. Stability Evaluation of CoMnOx@CG

**Figure 8:**
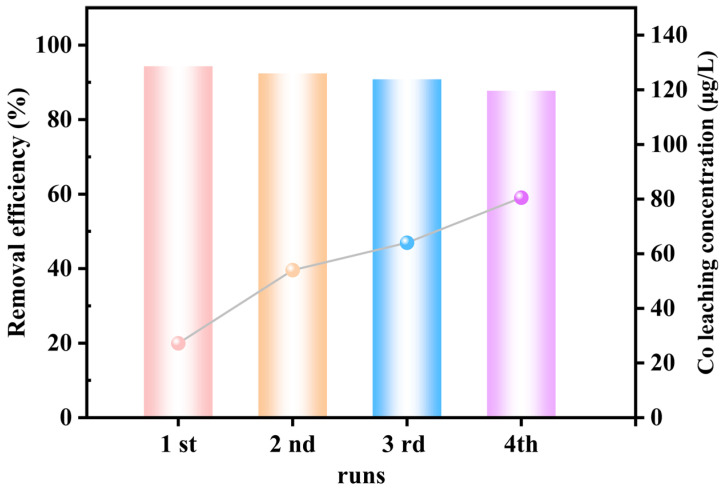
Representative phenol degradation performance of CoMnOx@CG/PMS over four consecutive cycles and the corresponding cobalt leaching in each cycle.

Stability assessment is essential for evaluating the practical application potential of the CoMnOx@CG/PMS system. As illustrated in Figure 8, the CoMnOx@CG catalyst exhibited excellent stability in phenol degradation, maintaining a removal efficiency above 85% after four successive cycles. The leached concentrations of Co and Mn were measured to be 80.5 and 74.8 μg/L, respectively, which are significantly lower than the permissible limits specified by GB 3838-2002 (Co = 1 mg/L, Mn = 0.1 mg/L) and prior studies (Figure S7 and Table 1). These results indicate negligible secondary pollution from the CoMnOx@CG catalyst during repeated use. The excellent stability and minimal metal leaching demonstrate that CoMnOx@CG possesses sustainable and stable catalytic activity, underscoring its potential for practical CCW applications. The consistently high degradation efficiency over multiple cycles, together with the very low Co and Mn leaching concentrations, suggests that the CoMnOx@CG catalyst retains its structural integrity and active sites during repeated PMS activation.

### 3.6. Degradation of Actual Coal Chemical Wastewater

**Figure 9:**
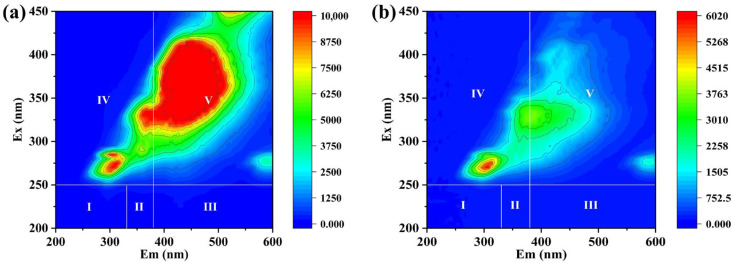
Three-dimensional excitation–emission matrix (3D-EEM) spectra of actual CCW: (**a**) before treatment and (**b**) after 60 min of reaction.

As shown in Figure 9a, the untreated wastewater exhibited strong fluorescence signals mainly in Regions IV and V, corresponding to soluble microbial by-product-like substances and humic-like macromolecules. The presence of these intense peaks indicates that the wastewater contains a large amount of highly conjugated and refractory organic matter with strong fluorescence characteristics. The collected coal chemical wastewater sample had an initial pH of 9.45 and an initial TOC concentration of 78.83 mg/L.

As shown in Figure 9 and Table S3, after being treated by the CoMnOx@CG catalytic system, the fluorescence peaks of the coal chemical wastewater in the three-dimensional fluorescence spectrum not only decreased significantly in intensity but also changed obviously in type. The fluorescence peaks of the coal chemical wastewater untreated by the CoMnOx@CG system were concentrated in regions IV and V, indicating that the wastewater contained high concentrations of dissolved microbial by-products and humic acid-like substances. After 60 min of treatment by the CoMnOx@CG system, the fluorescence intensity of the dissolved microbial by-products decreased significantly, and the fluorescence peaks of the humic acid-like substances almost disappeared, demonstrating that the CoMnOx@CG system has a good removal effect on the dissolved organic matter in the coal chemical wastewater, achieving TOC removal rate of 75.6%. Therefore, it can be seen that CoMnOx@CG has great potential in the application of catalyzing the PMS oxidation system to degrade coal chemical wastewater.

### 3.7. Degradation Pathways of Phenol in the CoMnOx@CG/PMS System

**Figure 10:**
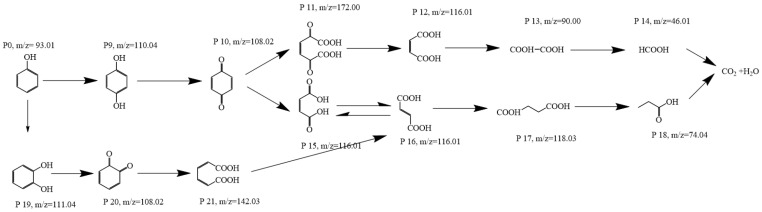
Degradation Pathways of Phenol in the CoMnOx@CG/PMS System.

In the CoMnOx@CG/PMS system, the degradation of phenol proceeds through a series of stepwise oxidation reactions involving ROS generated by PMS activation (Figure 10). These radicals attack the aromatic ring of phenol, initiating hydroxylation and ring-opening reactions that progressively break down the pollutant into smaller intermediates and, ultimately, fully mineralize it to CO_2_ and H_2_O [102,103].

Initially, the ROS attacks phenol’s aromatic structure to form hydroxylated intermediates such as catechol and hydroquinone [102]. These intermediates can undergo further oxidation to form quinone species, which are susceptible to ring-opening reactions that yield open-chain dicarboxylic acids and other fragmented organic acids. Continued oxidation of these low-molecular-weight carboxylic acids (e.g., maleic acid, fumaric acid, oxalic acid) results in further breakdown to CO_2_ and H_2_O, indicating deep mineralization [104].

## 4. Conclusions

In this study, a bimetallic Co–Mn oxide catalyst supported on coal gangue (CoMnOx@CG) was successfully synthesized via a simple wet-impregnation method and applied to activate PMS for phenol degradation under alkaline conditions. The CoMnOx@CG/PMS system exhibited high catalytic activity toward phenol removal in aqueous solution, achieving over 90% degradation within 10 min at pH 9. The catalyst also demonstrated good stability and reusability, maintaining phenol removal efficiencies above 85% after four consecutive cycles, with low Co and Mn leaching concentrations, indicating a low risk of secondary metal pollution. Mechanistic investigations based on quenching experiments and EPR analysis confirmed the simultaneous involvement of multiple reactive oxygen species (SO_4_·^−^, ·OH, and ^1^O_2_), suggesting a cooperative radical and non-radical oxidation pathway for phenol degradation. Furthermore, preliminary tests using real coal chemical wastewater samples showed a significant reduction in phenolic compounds and fluorescent dissolved organic matter, as evidenced by 3D-EEM and TOC analyses.

However, the practical applicability of the CoMnOx@CG/PMS system remains to be further validated, as long-term stability, degradation of a broader range of pollutants, and toxicity of degradation by-products were not fully assessed.

Therefore, while the results demonstrate the potential of CoMnOx@CG as a low-cost support for PMS activation catalysts, further comprehensive studies are required to fully evaluate its performance, mineralization efficiency, and environmental safety in complex wastewater systems.

## Figures and Tables

**Table 1:** Reported Cobalt Leaching from Various Catalysts in Previous Studies.

Solid Catalyst	Reaction Conditions	Leached [Co^2+^] (mg/L)	Reference
Co-Fe PBA	[catalyst] = 50 mg/L, [PMS] = 50 mg/L, pH = 3.5	0.2	[92,93]
Co_3_O_4_/NF	[catalyst] = 0.14 mg/L, [PMS] = 0.5 mM, pH = 3.0	0.015	[92,94]
CoFe_2_O_4_-Go	[catalyst] = 300 mg/L, [PMS] = 0.5 mM, pH = 7.0	0.1	[92,95]
Co-doped NaBiO_3_	[catalyst] = 200 mg/L, [PMS] = 1.0 mM, pH = 3.0	0.015	[96]
Co_3_O_4_	[catalyst] = 600 mg/L, [PMS] = 2.67 mM, pH = 7.0	0.73	[97]
CoFe_2_O_4_/TiO_2_	[catalyst] = 21 mg/L, [oxone] = 3.0 g/L, pH = 3.4	0.928	[98]
Chromate of copper and cobalt	[catalyst] = 200 mg/L, [PMS] = 0.5 mM, pH = 5.6	1.6	[99]
CoMn_2_O_4_-S	[catalyst] = 500 mg/L, [PMS] = 5.0 mM, pH = 3.26	2.46	[100]
Co_3_O_4_-Bi_2_O_3_	[catalyst] = 50 mg/L, [PMS] = 0.5 mM, pH = 3.4	0.043	[101]
CoFe_2_O_4_/TiO_2_	[catalyst] = 21 mg/L, [oxone] = 3.0 g/L, pH = 3.4	0.662	[98]
CoMnO_x_@CG	[catalyst] = 400 mg/L, [PMS] = 100 mg/L, pH = 9	0.08	This work

## Data Availability

The data presented in this study are available on request from the corresponding author.

## Supplementary Materials

The following supporting information can be downloaded at https://www.mdpi.com/article/10.3390/toxics14010029/s1, Figure S1: Schematic illustration of the synthesis process for CoMnOx@CG; Figure S2: SEM images of coal gangue; Figure S3: Elemental mappings of coal gangue; Figure S4: The EDS spectra of coal gangue; Figure S5: XPS spectra of survey for CoMnOx@CG; Figure S6: The XPS spectra of the spent CoMnOx@CG catalyst: (a) Mn 2p and (b) Co 2p regions; Figure S7: Mn leaching; Table S1: Elemental composition obtained from EDS mapping; Table S2: Comparison of catalytic activity on various catalysts for phenol degradation. Table S3: The boundary of the fluorescence spectral region. References [105,106,107,108,109,110,111,112,113,114,115,116] are cited in the Supplementary Materials.
