# Supplementary material for: Synergistic Activation of Peroxymonosulfate by CoMnOx Supported on Coal Gangue for Alkaline Wastewater Treatment
Source: Toxics. 2025 Dec 26;14(1):29. doi: 10.3390/toxics14010029 (PMC12845686; doi:10.3390/toxics14010029)
Supplement: Supplementary file 1 [file toxics-14-00029-s001.zip › toxics-4025864-supplementary.pdf]

Supplementary Material

Synergistic Activation of Peroxymonosulfate by CoMnOx Supported on Coal Gangue for Alkaline Wastewater Treatment

Ke An [1], Weiwei Yang [1,*], Houhu Zhang [2,*]

[1] School of Chemical and Environmental Engineering, China University of Mining and Technology-Beijing, Beijing 100083, China
[2] Nanjing Institute of Environmental Sciences, Ministry of Ecology and Environment of the People's Republic of China, Nanjing 210042, China
* Correspondence: wwycumtb@163.com (W.Y.); zhanghouhu2008@163.com (H.Z.)

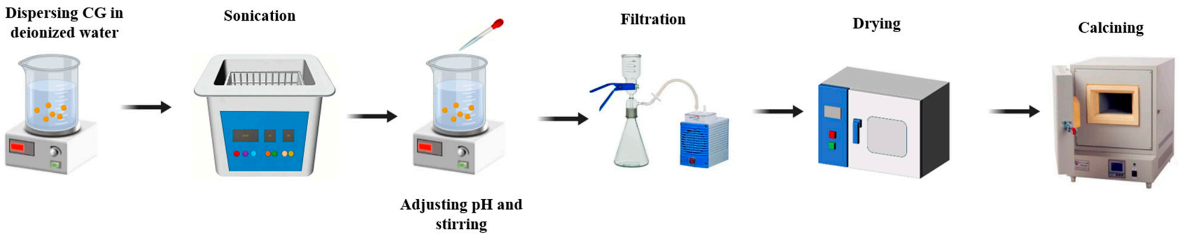

Figure S1. Schematic illustration of the synthesis process for CoMnOx@CG

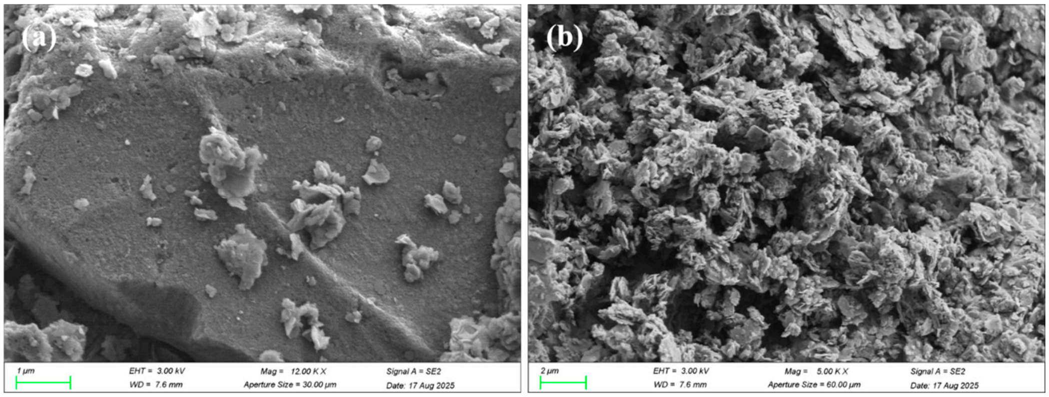

Figure S2. SEM images of coal gangue

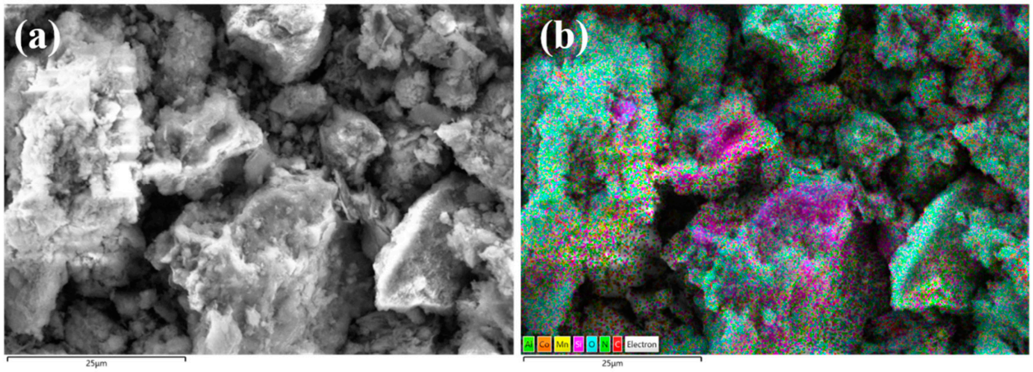

Figure S3. Elemental mappings of coal gangue.

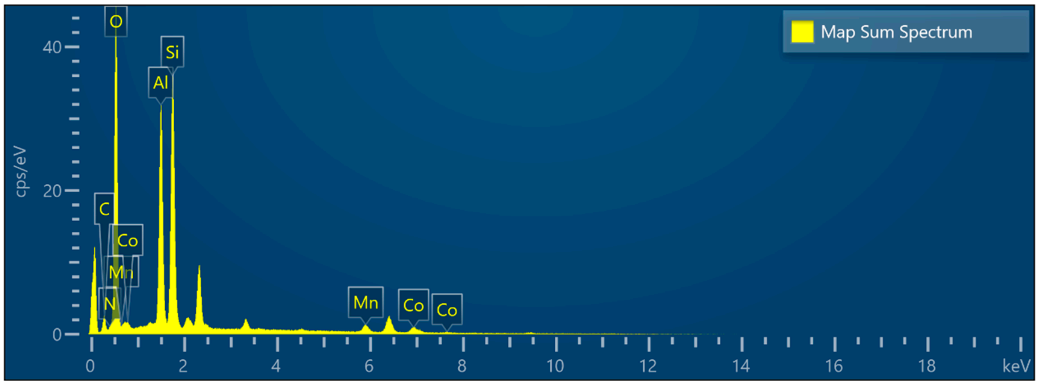

Figure S4. The EDS spectra of coal gangue.

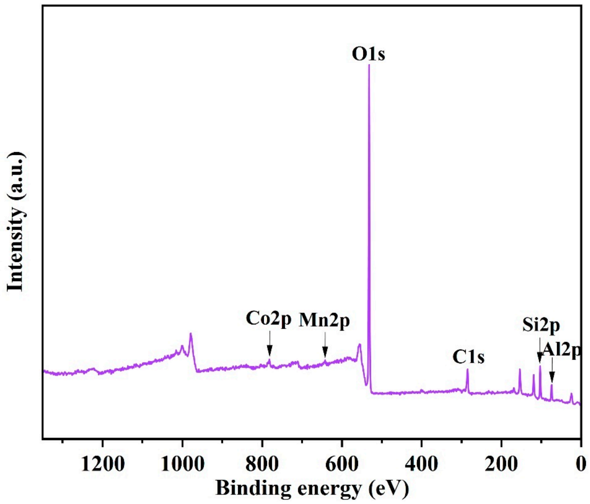

Figure S5. XPS spectra of survey for $CoMnO_x$@CG

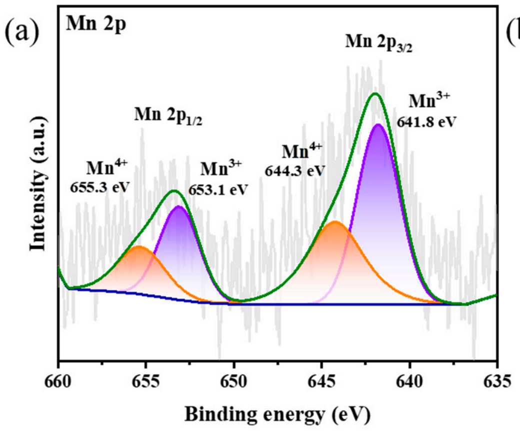

Figure S6. The XPS spectra of the spent $CoMnO_x$@CG catalyst: (a) Mn 2p and (b) Co 2p regions.

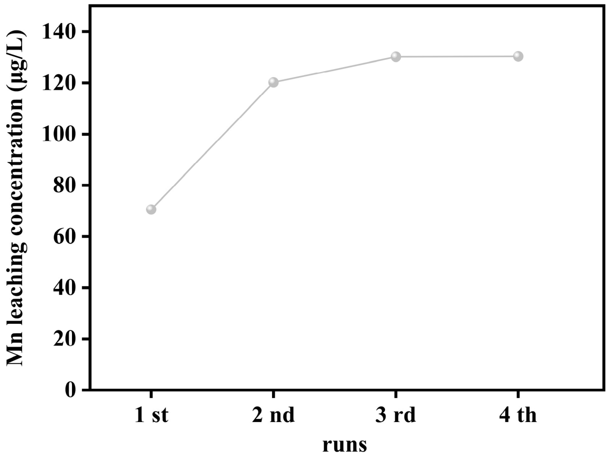

Figure S7. Mn leaching.

Table S1. Elemental composition obtained from EDS mapping

Element	Line Type	Weight %	Weight % Sigma	Atomic %
C	K series	13.87	0.42	21.33
N	K series	0.35	0.34	0.47
O	K series	47.22	0.33	54.50
Si	K series	18.40	0.15	12.09
Mn	K series	2.95	0.12	0.99
Co	K series	3.12	0.17	0.98
Al	K series	14.10	0.12	9.65
Total		100.00		100.00

Table S2. Comparison of catalytic activity on various catalysts for phenol degradation.

Catalyst	[Phenol]	[Persulfates]	Rate constant (min^{-1})	TOC removal (%)	Reference
Porous MnO_2	20 mg/L	PMS-2 g/L	0.0869		[105]
$BioMnO_x$	30 mg/L	PMS-1 g/L	0.105	89.56%	[106]
$LaCo_{0.5}Mn_{0.5}O_{3+\delta}$	20 mg/L	PMS-2 g/L	0.052	67%	[107]
$LaCoO_3$	10 mg/L	PS-10 mM	0.029		[108]
$CeVO_4$	100 mg/L	PMS-2 g/L	0.0205	＞ 90%	[109]
Fe(II)-doped g-C_3N_4	0.1mM	PMS-5 mM	0.183		[110]
CuMgFe-LDO	0.1mM	PS-0.5 g/L	0.102		[111]
1T/2H-MoS_2/$CuFe_2O_4$	20 mg/L	PMS-1 mM	0.15353		[112]
TiO_2 nanotube arrays	100 μM	PMS-1mM	0.0488	~70%	[113]
$FeCo_2O_4$	0.53 mM	PMS-3 mM	0.15	36%	[114]
OMS-2	20 mg/L	PMS-0.15 g/L	0.22518	85.2%	[115]
MoS_2	10 mg/L	PMS-0.5mM	0.196	75%	[116]
CoMnOx@CG	20 mg/L	PMS-0.1	0.26	75.6%	This

	g/L	work

Table S3. The boundary of the fluorescence spectral region.

Region	Types of organic substances	Excitation wavelength Ex (nm)	Emission wavelength Em (nm)
I	Aromatic protein-like substances I	200～250	280～330
II	Aromatic protein-like substances II	200～250	330～380
III	Substances of fulvic acid type	200～250	380～500
IV	Dissolved microbial metabolites	250～380	280～380
V	Substances of humic acid type	250～400	380～550

References

105. LIU Q R, DUAN X G, SUN H Q, et al. Size-Tailored Porous Spheres of Manganese Oxides for Catalytic Oxidation via Peroxymonosulfate Activation [J]. Journal of Physical Chemistry C, 2016, 120(30): 16871-8.
106. TIAN N, TIAN X K, NIE Y L, et al. Biogenic manganese oxide: An efficient peroxymonosulfate activation catalyst for tetracycline and phenol degradation in water [J]. Chem Eng J, 2018, 352: 469-76.
107. MIAO J, SUNARSO J, DUAN X G, et al. Nanostructured Co-Mn containing perovskites for degradation of pollutants: Insight into the activity and stability [J]. J Hazard Mater, 2018, 349: 177-85.
108. MANOS D, PAPADOPOULOU F, MARGELLOU A, et al. Heterogeneous Activation of Persulfate by $LaMO_3$ (M=Co, Fe, Cu, Mn, Ni) Perovskite Catalysts for the Degradation of Organic Compounds [J]. Catalysts, 2022, 12(2): 187.
109. OTHMAN I, ZAIN J H, ABU HAIJA M, et al. Catalytic activation of peroxymonosulfate using $CeVO_4$ for phenol degradation: An insight into the reaction pathway [J]. Applied Catalysis B-Environmental, 2020, 266.
110. FENG Y, LIAO C Z, KONG L J, et al. Facile synthesis of highly reactive and stable Fe-doped g-C_3N_4 composites for peroxymonosulfate activation: A novel nonradical oxidation process [J]. J Hazard Mater, 2018, 354: 63-71.
111. CHEN Y, YAN J C, OUYANG D, et al. Heterogeneously catalyzed persulfate by CuMgFe layered double oxide for the degradation of phenol [J]. Applied Catalysis a-General, 2017, 538: 19-26.
112. DING S Y, REN X C, CHEN R H, et al. Efficient degradation of Phenol by 1 T/2H-MoS_2/$CuFe_2O_4$ activated peroxymonosulfate and mechanism research [J]. Applied Surface Science, 2023, 612.
113. YOO H Y, KIM M S, SHIN H, et al. Peroxymonosulfate activation by black TiO2 nanotube arrays under solar light: switching the activation mechanism and enhancing catalytic activity and stability [J]. J Hazard Mater, 2022, 433: 128796.
114. WANG C, ZHAO J Y, CHEN C M, et al. Catalytic activation of PS/PMS over Fe-Co bimetallic oxides for phenol oxidation under alkaline conditions [J]. Applied Surface Science, 2021, 562.
115. WEI J, LI X, YANG Q, et al. Sulfate radical-mediated degradation of phenol and methylene blue by manganese oxide octahedral molecular sieve (OMS-2) activation of peroxymonosulfate [J]. Environmental Science and Pollution Research, 2019, 26(13): 12963-74.
116. DU M M, YI Q Y, JI J H, et al. Sustainable activation of peroxymonosulfate by the Mo(IV) in MoS_2 for the remediation of aromatic organic pollutants [J]. Chinese Chemical Letters, 2020, 31(10): 2803-8.
